# Care Cascades for Hypertension in Low-Income Settings: A Systematic Review and Meta-Analysis

**DOI:** 10.3389/ijph.2023.1606428

**Published:** 2023-10-12

**Authors:** Demetrio Lamloum, Federico Fassio, Brianna Osetinsky, Fabrizio Tediosi

**Affiliations:** ^1^ Department of Preventive, Restorative and Pediatric Dentistry, University of Bern, Bern, Switzerland; ^2^ Department of Public Health, Experimental and Forensic Medicine, University of Pavia, Pavia, Italy; ^3^ Department of Public Health, Experimental and Forensic Medicine, Section of Biostatistics and Clinical Epidemiology, University of Pavia, Pavia, Italy; ^4^ Department of Epidemiology and Public Health, Swiss Tropical and Public Health Institute, Basel, Switzerland; ^5^ University of Basel, Basel, Switzerland

**Keywords:** hypertension (HTN), care cascade, low income country (LIC), lower-middle income country (LMIC), prevalence

## Abstract

**Objective:** High blood pressure is the leading risk factor for cardiovascular disease. The hypertension care cascade (HCC) is increasingly being used to evaluate the effectiveness of interventions. This systematic review aims to examine HCC in low-income settings.

**Methods:** The search strategy included articles published between January 2010 and April 2023. We excluded studies with incomplete HCC, on fragile patients or aged <18 years, reviews. We used the MOOSE guideline. Five researchers retrieved data on the survey year, country, population, HCC and diagnostic methods for hypertension. We used JBI Critical Appraisal Tools for quality assessment.

**Results:** Ninety-five articles were analyzed. Average hypertension prevalence was 33% (95% CI: 31%–34%), lower in LICs than in LMICs (25% vs. 34%). The overall mean awareness of hypertension was 48% (95% CI: 45%–51%), its treatment was 35% (95% IC: 32%–38%) and its control 16% (95% CI: 14%–18%). In almost all steps, percentages were lower in LICs and in Sub-Saharan Africa.

**Conclusion:** Trends in HCC vary between countries, with poorer performance in LICs. This review highlights the need for interventions tailored to low-income settings in order to improve hypertension care.

## Introduction

Cardiovascular diseases (CVDs) are the main cause of illness, death, and disability worldwide. They were responsible for approximately 393 million (95% UI 368–417) disability adjusted life years (DALYs) in 2019 and were the leading cause of disease burden, accounting for 18.6 million (17.1–19.7) deaths. Raised blood pressure is the preeminent risk factor for CVDs, contributing to 235 million (95% IU 211–261) DALYs and 10.8 million (9.51–12.1) deaths. Overall, 8.5 million people died due to hypertension, 88% in Low (LIC) and Lower Middle-Income Countries (LMICs) [1, 2]. In LICs and LMICs, increases in the four main socio-economic related behavioral risk factors for hypertension, namely physical inactivity, unhealthy diet, tobacco use, and alcohol consumption, are strongly linked to the soaring hypertension epidemic [3–5]. While hypertension can be effectively managed at the primary care level through a combination of lifestyle changes, counselling, and pharmaceutical interventions, it is evident that many health systems in low income settings are ill-equipped to handle this condition [6–11]. According to the Lancet Commission on Hypertension and the World Heart Federation, controlled hypertension in affected patients are key indicators for health systems, and leads to reduced associated disability and premature mortality, in addition to avoiding substantial productivity losses [12, 13]. However, effective hypertension management relies on raising awareness about the condition and ensuring active engagement in appropriate treatment. The cascade of care, which encompass the journey from diagnosis to control, serves as a valuable framework to evaluating health systems, particularly in the context of chronic diseases [14, 15].

From 2000 to 2010, the rates of hypertension diagnosis, awareness, treatment, and control improved by ≥10% in High Income Countries. By contrast, in LICs and LMICs, improvements were narrow for awareness (32.3%–37.9%), treatment (24.9%–29.0%), and control (8.4%–7.7%) [16]. Hence, a deep dive into hypertension cascade of care is pivotal to define a standard for monitoring and planning public health strategies to manage hypertension control [17]. Despite extensive current evidence providing pooled analysis about hypertension care cascades across different continents, none appear to spotlight LICs and LMICs [1, 18]. Compared to both High and Upper-Middle Income countries, LICs and LMICs had higher age standardized mortality rates from high blood pressure and related cardiovascular diseases [16].

To effectively combat the mortality and morbidity associated with hypertension, it is imperative to deepen our understanding of disparities within the hypertension care continuum. This knowledge forms a crucial foundation for enhancing both national and international initiatives aimed at addressing uncontrolled hypertension. Quality improvement methodologies such as Plan-Do-Study-Act cycles, cascade analysis, process mapping, root cause analysis offer valuable tools for advancing our efforts in this critical endeavor [19].

Therefore, the objective of this study is to analyze disparities in hypertension prevalence, awareness, treatment, and control across various factors such as income level, geographical area, survey year, and population size.

## Methods

### Search Strategy and Inclusion Criteria

The review process was developed in accordance with Meta-analysis Of Observational Studies in Epidemiology (MOOSE) Guidelines [20]. It comprised searches from four electronic databases—PubMed, Embase, Scopus, and CHINAL Complete. The research protocol was uploaded on PROSPERO (ID CRD42022339717). The collected studies were published between January 2010 and April 2023, concerning hypertension care cascade in LICs and LMICs according to the World Bank classification by income level [21] (Supplementary Material S1). A selected combination of key words and MeSH terms coupled to hypertension care cascade were applied. We collected studies that included all of the following to inform the full hypertension care cascade: prevalence, awareness, treatment, and control for hypertension (Supplementary Material S2). The process of study selection comprised two-phases. First, search results were imported into Rayyan (https://www.rayyan.ai/) to manage citations. Second, two researchers conducted a title-abstract screening taking into consideration prevalence, awareness, treatment, and control of hypertension. A third reviewer solved conflicts. Studies not reporting a full care cascade for hypertension or with inconsistent data for prevalence and/or awareness and/or treatment and/or control of hypertension were excluded. Articles in English, French, Spanish, and Italian were included. Multiple years within the same studies on the same country were included if presenting clear data by year. Reviews, meta-analyses, comments, editorials, studies focusing on patients’ subgroups (e.g., chronic kidney disease, chronic cardiovascular disease), study settings that could embed selection bias (e.g., universities, hospitals), patients affected by specific diseases other than hypertension, or undergoing medical treatments for those conditions, or paediatric population (<18 years) were excluded.

### Data Extraction

Five independent researchers conducted data extraction that included: the title, author/s, publication year, journal, survey year, country of study, country-income classification, sample size, study population, age range, diagnostic methods for hypertension, and information regarding the prevalence, awareness, treatment, and control of hypertension. Data about risk factors (e.g., smoking), BMI, diabetes, sex, age mean, or median were also retrieved when present. When absolute numbers were not available for care cascade steps, we retrieved them from unweighted frequency rates. Authors were contacted in case of discordant or not clearly reported data.

### Quality Assessment

A cross-sectional assessment of the risk of bias was conducted using the Joanna Briggs Institute (JBI) Critical Appraisal Tools. Discrepancies were resolved through a two-round assessment process [22]. The specific criteria of JBI checklists can be found in the Supplementary Material (JBI Checklist).

### Definition of Hypertension, Awareness, Treatment, and Control

The available studies use different definitions and different denominators to present their results, thus requiring a standardized way to present our results. Most of the studies employed a definition of hypertension as a systolic blood pressure (SBP) greater than or equal to 140 mmHg and/or a diastolic blood pressure (DBP) greater than or equal to 90 mmHg, or self-reported use of antihypertensive medication.

Awareness was measured as self-reported prior diagnosis of hypertension and expressed as the proportion of individuals with hypertension who were aware of their condition. Treatment was defined as the use of medication to manage hypertension at the time of the study and was reported either among all individuals with hypertension or among those who were aware of their condition. Control was defined as an SBP less than 140 mmHg and a DBP less than 90 mmHg, and was reported among all individuals with hypertension, those who were aware of their condition, or those receiving treatment for hypertension.

### Data Analysis

The analysis of the cascade of care is based on cross-sectional studies and longitudinal studies with a cross-sectional data analysis. We considered four stages of the hypertension continuum of care which correspond to prevalence, awareness, treatment, and control. This approach focuses on identifying the losses that occur when patients move from one stage to another. In addition to loss between stages, we analyzed each stage of awareness, treatment, and control as a proportion of the population with hypertension. We used the metaprop package (STATA17) applying the Freeman Tukey transformation [23] and exact method for confidence intervals. We used a random effect model to consider differences across countries. Subgroups analysis considered Gross National Income (GNI), geographical area and time period before-after 2015 [24]. The I^2^ statistic was used to evaluate differences between subgroups. To test the results robustness, we conducted sensitivity analysis not considering May Measurement Month (MMM) studies as subjects are volunteers, or studies reporting only cascade step percentages or excluding studies with a high risk of bias (JBI score <5). We also performed sensitivity analysis excluding studies conducted in India, due to high number of publications, and excluding studies with an ill-defined survey date or a follow-up longer than 2 years. Since predictor variables (i.e., smoke habits, diabetes, BMI age) were not available for all the studies, we did not run any meta-regression.

### Publication Bias

We used doiplot for publication bias, considering prevalence of hypertension as effect size [25], the LFK index to test statistical significance, and JBI qualitative evaluation to identify risk of bias [26]*.* We considered a JBI score <5 as high risk of bias, 5–6 as moderate and 7–9 as low.

## Results

Through the search strings (Supplementary Material S2) 3,889 articles were retrieved. After references imported in Zotero and Rayyan, 1,043 articles were removed as duplicates. Following title and abstract analysis, 2,630 papers were excluded, failing to meet the inclusion criteria. 216 were eligible for full text analysis and 95 were eventually included for data extraction (Supplementary Figure S1). [27–121] Three studies had data about more than one population.

Eventually, 100 cascades of care were analysed (Supplementary Material S3). 16 studies were conducted in East Asia & Pacific, 36 in Sub-Saharan Africa, 31 in South Asia, 13 studies in Middle East & North Africa, 3 studies in Latin America & Caribbean and 1 in Europe & Central Asia. Among the included articles, 31 were MMMs. After the JBI quality assessment, 40 studies (including all the MMMs) were at high risk of bias, 20 at moderate, and 40 at low risk (Supplementary Material S4). Data on age, BMI, gender, diabetes, and smoking habits were not available for all articles (Supplementary Material S5).

### Hypertension Prevalence

**FIGURE 1:**
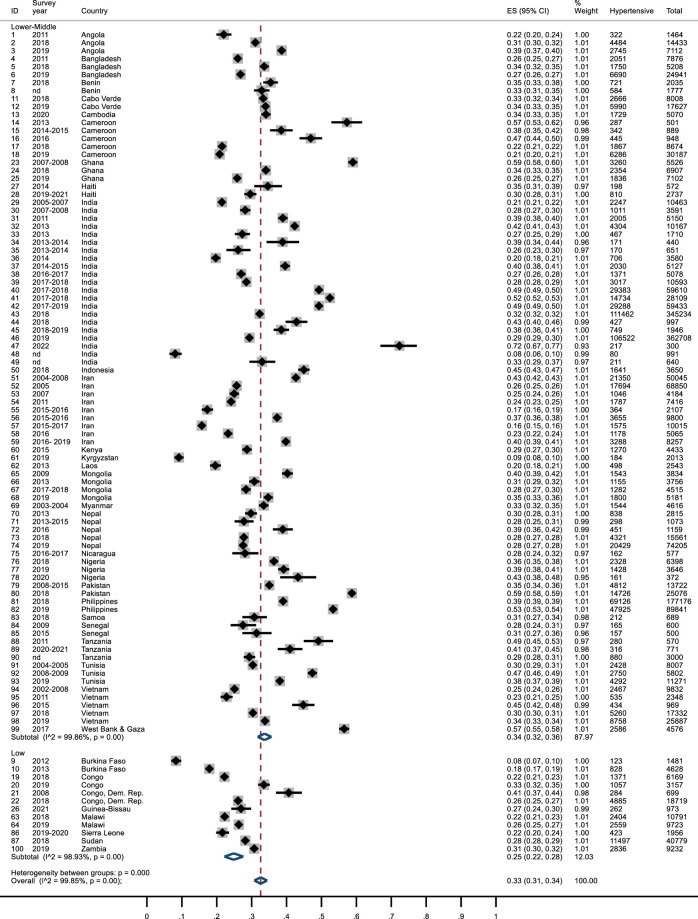
Forest plot of hypertension prevalence by country income. The effect size (ES) ranges from 0 to 1. The survey year refers to the year when the survey was conducted. The correspondence between the identification number (ID) and the reference is reported in Supplementary Table S3. Lower Income countries and Lower-Middle Income Countries, January 2010 and April 2023.

The overall prevalence of hypertension was 33% (95%CI: 31%–34%) (Figure 1). Six studies found a prevalence higher than 50% (Pakistan [101] and Ghana [48], 59%; Gaza and Cameroon [120], 57%; Philippines [103], 53%; India [66, 72], 72% and 52%). In three studies, hypertension prevalence was lower than 10% (Kyrgyzstan [84], 9%; Burkina Faso [35] and India [73], 8%). The doiplot graph suggested no publication bias (Supplementary Figure S2). The subgroup analysis for geographical area (Supplementary Figure S3) showed slight difference between geographical areas. Hypertension prevalence was lower in LICs than LMCIs (25% vs*.* 34%; *p* <0.001) as shown in (Figure 1). No significant differences in study results were observed when comparing data from studies conducted before and after 2015, as indicated in (Supplementary Figure S5). Furthermore, excluding studies with an ill-defined or excessively long survey date did not yield any discernible differences, as shown in (Supplementary Figure S4). Additionally, four studies reported hypertension data as percentage, an conducting a sensitivity analysis that excluded these articles resealed no significant variations, as depicted in (Supplementary Figures S6, S7).

After excluding MMMs or studies with a high JBI risk of bias, the overall prevalence was similar to the comprehensive analysis, while confidence intervals were slightly wider, and the risk of publication bias seemed to increase (Supplementary Figures S8–S11). Percentage did not change after removing the studies conducted in India (Supplementary Figures S12, S13).

### Awareness

**FIGURE 2:**
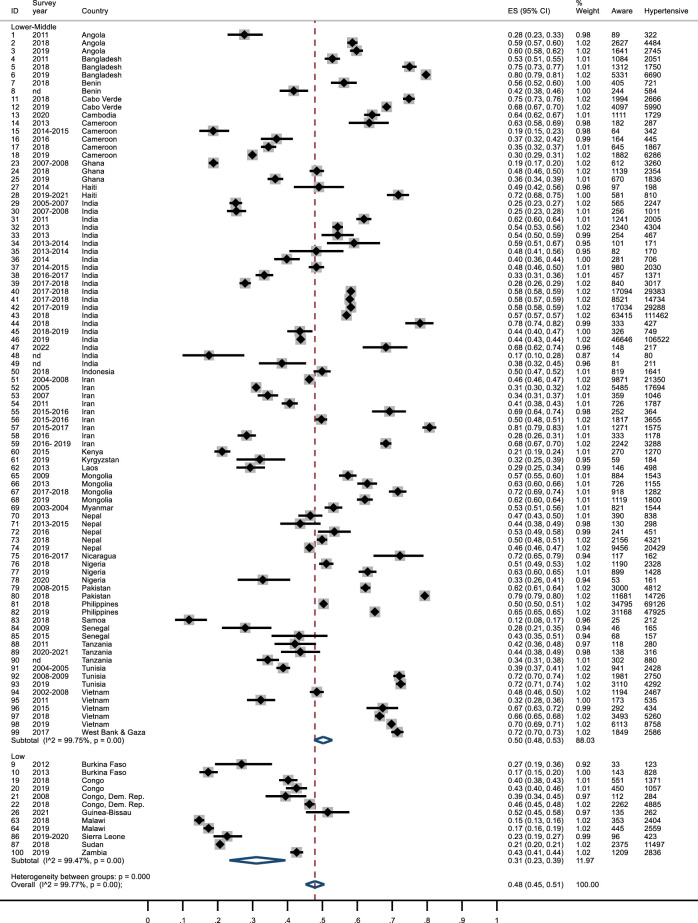
Awareness of hypertension forest plot by country income. Lower Income countries and Lower-Middle Income Countries, January 2010 and April 2023.

The overall mean awareness among hypertensive subjects was 48% (95% CI: 0.45–0.51). In seven studies it was less than 20% (Burkina-Faso [36], Cameroon [41], Ghana [49], India [73], Malawi [86, 87] and Samoa [104]), while in four surveys is was higher than 75% (Bangladesh [32], India [69], Iran [80] and Pakistan [101]). Awareness was lower in LICs (0.31; 95% CI: 0.23–0.39) compared to MICs (0.50; 95% CI: 0.48–0.53) Figure 2. In Sub-Saharan Africa, 39% of subjects were aware of hypertension, less than in Middle East and North Africa (0.54; 95% CI: 0.45–0.64). In East Asia & Pacific and South Asia, awareness was around 50% (0.54; 95% CI: 0.49–0.59), whereas in Latin America & Caribbean was higher (0.65; 95% CI: 0.50–0.78) (Supplementary Figure S14). After 2015, awareness of hypertension appeared to have increased (Supplementary Figure S16). After excluding publications with data about awareness reported as percentage, MMMs, studies with JBI score<5 or reports from India, the values relating to the awareness of hypertension did not change significantly (Supplementary Figures S15, S17–S20).

### Treatment

**FIGURE 3:**
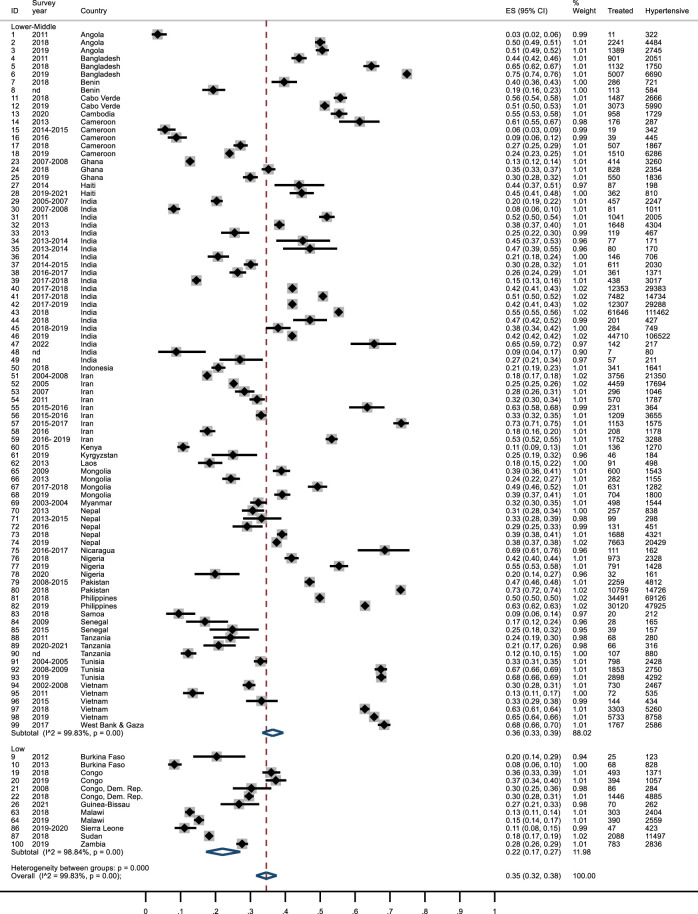
Forest plot: treatment of hypertension by income. Lower Income countries and Lower-Middle Income Countries, January 2010 and April 2023.

Overall, 35% of hypertensive subjects were in treatment (95% IC: 0.32–0.38). Estimates ranged from 3% in Angola [27] to over 70% in Bangladesh [32], Iran [80]and Pakistan [101], although about half of the studies found a percentage of treated subjects between 20% and 50%. Treatment of hypertension was higher in LMICs (0.36; 95% CI: 0.33–0.39) than in LICs (0.22; 95% CI: 0.17–0.27) (Figure 3). There were differences across geographical areas: in Sub-Saharan Africa treatment rates were lower compared to Middle East & North Africa (26% vs*.* 44%). In Asia results were similar (South Asia 0.39; 95% CI: 0.35–0.43; East Asia & Pacific 0.37; 95% CI: 0.31–0.43). In Latin America almost half of hypertensive patients were treated (Supplementary Figure S21).

Treatment of hypertension seemed to have increased after 2015 (Supplementary Figure S23). The overall percentage of treated subjects among those aware of having hypertension was 74% (95% CI: 0.70–0.78), with no difference between LICs and LMICs (Supplementary Figure S24) and some differences across geographical areas (Supplementary Figure S25). After removing 13 studies with treated subjects reported as a percentage, results were similar (Supplementary Figure S26). After excluding the MMMs studies, the overall percentage decreased from 35% to 31% (Supplementary Figure S27). Similar results emerged after excluding studies with a JBI score <5 (Supplementary Figure S28). Percentages did not change after removing results from India (Supplementary Figure S29).

### Control

**FIGURE 4:**
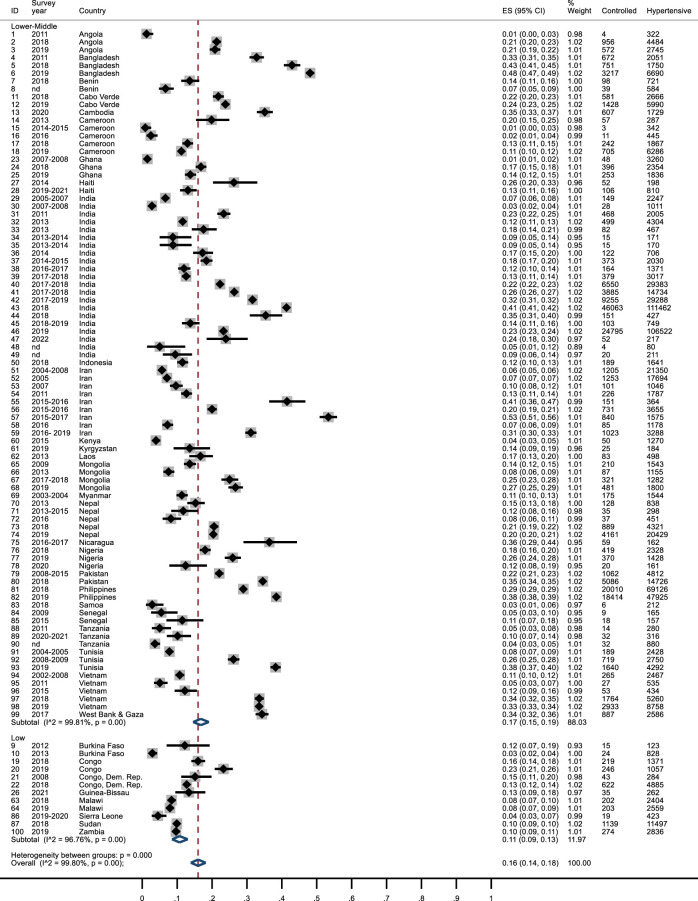
Forest plot: control of hypertension by income. Lower Income countries and Lower Middle-Income Countries, January 2010 and April 2023.

Overall, 16% of hypertensive subjects had their hypertension controlled (95% CI: 0.14–0.18). Three studies reported a control of hypertension <2% (Angola [27], Cameroon [41] and Ghana [49]), whereas two studies in Bangladesh [31, 32], one in India [68] and two in Iran [78, 80] found >40% of controlled hypertensive subjects. In LICs the percentage of controlled hypertension was lower than LMICs (11% vs*.* 17%) (Figure 4). Studies conducted in Sub-Saharan Africa showed a percentage below the global mean (0.11; 95% CI: 0.09–0.13), while the other geographical areas had higher values (Supplementary Figure S30). After excluding MMMs studies or those with a JBI score <5, the percentage of controlled hypertension reduced to 14% (Supplementary Figures S32, S33). Control of hypertension was higher in more recent studies (Supplementary Figure S34). Results did not change after removing studies from India or those with data reported as percentages or studies with ill-defined survey date (Supplementary Figures S31, S35, S36). Subjects with controlled hypertension, among those being treated, were about 47% (95% CI: 0.45–0.50), without significant differences between LICs and LMICs (Supplementary Figure S37). Four studies reported a score <20% (Cameroon [41] 16%; Ghana [49] 12%; India [60, 61] 19%), while in 3 studies >80% of treated subjects were controlled (India [62, 64] 84% and 87%; Laos [85] 91%). Sub-Saharan Africa showed a percentage of treated subjects below the global mean (42%) (Supplementary Figure S38).

## Discussion

To our knowledge, this study represents the first comprehensive examination of the complete hypertension cascade of care specifically focused on LICs and LMICs. The findings offer valuable insights into the global challenge of hypertension in resource-constrained settings, revealing significant variations across countries and geographical regions. We collected data from 35 countries, representing a total population of 1.5 billion individuals.

Our study highlights the overall unpreparedness of the examined health systems, with only a small percentage of individuals with hypertension being aware of their condition and receiving treatment, and an even lower percentage achieving control over their hypertension. Although positive results in hypertension control rates were observed during the 80s and 90s, it has since plateaued, raising concerns about a potential decline in progress [122].

These findings underscore the pressing need for enhanced hypertension care and management, particularly in low-income settings. Supporting the idea that LICs and LMICs should be considered separately, our analysis revealed that LMICs outperformed LICs in all stages of the cascade of care. This is consistent with previous research, emphasizing that hypertension poses a growing challenge regardless of the income level [1]. Significant gaps exist among countries and especially across geographical areas, [1, 104]. East Asia and the Pacific region exhibited the highest prevalence rates at 32%, whereas South Asia had the lowest rates at 24%. Conversely, Latin America and the Caribbean demonstrated better performance in terms of awareness, treatment, and control, while Sub-Saharan African countries showed lower scores. Our sensitivity analysis confirmed that the limited data from Latin America and the Caribbean did not unduly influence our overall results. Social determinants of health play a crucial role in noncommunicable diseases [123]. Addressing the hypertension epidemic requires a multidisciplinary and multilateral approach to prevent further widening of health gaps, particularly in more fragile countries [124]. Lower levels of education and/or income are risk factors associated with higher dropout rates from the care pathway [125]. Moreover, inadequate patients’ preparedness may impede understanding of health information and limit patients’ engagement. Out of pocket payments and opportunity costs remain a significant barriers to accessing care [126], exacerbated by staff shortage. Removing or lowering user fees and investments in mid-level providers in hypertension management in low-income settings are both important policies to improve access to care [127, 128].

Prioritizing health policies that specifically target hypertension care in vulnerable populations can effectively reduce disparities in healthcare access and mitigate the risk of catastrophic health expenditures [129]. Moreover, the implementation of a quality improvement collaborative approach to promote the adoption of hypertension (HTN) care cascade and its integration with communicable disease programs, notably HIV, has demonstrated noteworthy effectiveness in the short term while also presenting favorable long-term prospects, as underscored in the study by Basenero et al. [19].

However, it is important to note that, even when health systems are optimally organized, simply ensuring access may not be sufficient to improve population health [130]. Quality standards, such as ensuring the right patient receives care in the right place and at the right time [131] are essential components that must be incorporated to achieve significant improvements in overall population health outcomes.

In contrast to the recent study conducted by the NCD Risk Factor Collaboration (NCD-RisC) [1], our review focused exclusively on peer-reviewed publications available in prominent scientific databases, with the deliberate exclusion of studies from grey literature. Despite the presence of numerous grey literature publications (*i.e.* World Bank reports), we decided to exclude it. Several compelling reasons informed this choice. First of all, it should be note that the WHO reports are themselves based on the MMM studies, which were the included in our investigation. Furthermore, our rigorous analysis and inclusion criteria demanded a stringent assessment of cluster definitions within the studies considered dictated by the cascade of care model. This involved the comprehensive consideration of all stages of the care cascade and the examination of general populations rather than specific clusters (i.e. HIV, CKD, etc.). In order to ensure the robustness of our findings, sensitivity analyses were conducted to confirm that the exclusion of specific items did not significantly impact the final results [132].

Additionally, we observed a growing trend of policies aimed at reducing the burden of cardiovascular diseases, leading to an increase in national and sub-national surveys as well as the development of technical packages [74]. This trend, however, results in multiple publications for the same country, which, for the purpose of our study, appeared inconsistent and-or redundant. Even hypertension prevalence-based studies and large courts surveillance programs that did not fit our inclusion criteria are pivotal to implement public health policies and scarce healthcare resources’ management [133, 134].

A distinctive advantage of this review lies in its comprehensive examination of the entire care cascade, coupled with the differentiation between, LICs and LMICs as distinct entities. This approach not only facilitates the assessment of disease control but also enables the identification of critical steps within the cascade where a significant proportion of individuals may be lost, thus offering valuable insights to inform the development of interventions and future policy initiatives [110].

Furthermore, the identification of dropout rates at each stage of the cascade has the potential to highlight the need to expand the scope of comprehensive, multidimensional interventions, particularly at the primary care level, with the aim of improving overall health outcomes.

One of the limitations we encountered pertains to the diverse methods employed for blood pressure measurement in certain studies, although it’s worth highlighting that, in the majority of cases, the WHO guidelines were adhered to. However, it is crucial to emphasize that all the studies we included into our analysis uniformly embraced the internationally recognized parameter of a fixed threshold for diagnosing hypertension, specifically set at 140–90 mmHg [135].

A validated automated sphygmomanometer was used in all studies, but digital (OMRON) sphygmomanometers were not systematically applied. Additionally, paper-based questionnaires may have introduced bias in data recording. These disparities may have affected some of the outcomes observed across countries and regions. However, key features in the questionnaires regarding individuals’ history of hypertension diagnosis and hypertensive of medication use were comparable. As already stated, we considered pregnant women and patients affected by other chronic conditions (i.e., kidney or infectious disease) as potential bias for the results, so we excluded studies focusing on these populations from our analysis.

Although we did not conduct a temporal analysis due to insufficient data, it is worth noting that since 2015, some countries have shown signs of improvement in terms of awareness, treatment, and control of hypertension. These positive changes may be attributed to local advocacy efforts and awareness campaigns, highlighting the potential for enhanced hypertension care even in resource-constrained settings [43, 44].

The hypertension cascade of care steps analyzed in this study did not include information about gender and other variables due to limited reporting. We were only able to provide an average representation of the female population, which comprised approximately 50% of the total participants interviewed across the studies. However, it’s worth noting that in some studies, this percentage ranged from as low as 25% to as high as 70%.

In less than half of the studies included in our analysis, we could readily extract specific data on individuals with diabetes, smokers, or those who were overweight. Notably, when it came to overweight, many studies primarily reported data only for obese subjects (BMI≥30). Moreover, there was uneven representation of countries, and methodological differences could led to heterogeneous results. To address these limitations, subgroup analyses were performed. Additionally, the JBI quality scores for some studies were found to be low.

MMM studies constitute a significant component of the meta-analysis basket of publications. Despite the limitations associated with recruiting voluntary participants, which may result in non-representative sampling, these surveys offer valuable insights for understanding global trends more accurately. Incorporating MMM studies in the analysis allows for a more comprehensive examination and enables stakeholders to better understand changes in care cascades over time.

Lastly, it is important to highlight that while the current care cascade framework includes outcome quality measures, it regrettably lacks any specific mentions pertaining to the quality of health services. The omission of quality-based indicators is notable because it restricts the comprehensive analysis of health system intervention performance. The inclusion of quality-based measures in the evaluation framework is essential to gain a more holistic understanding of the effectiveness of interventions. It allows for a nuanced assessment of not only outcomes, but also the processes and structures that underpin the delivery of healthcare. By overlooking the quality of health services, we risk an incomplete assessment of health interventions, hindering our ability to make informed decisions and improve health systems [132].

### Conclusion

This study further substantiates the escalating issue of hypertension in LICs and the consistent disparities observed when compared to LMICs. It is imperative to enhance awareness regarding the prevalence, treatment, and control of hypertension among the population, particularly in LICs, to facilitate adequate investment in the management of this disease. This cross-country comparison study, focusing on hypertension care cascades, underscores the critical importance of conducting comprehensive and rigorous assessments of the entire spectrum of hypertension care. Employing standardized methods ensures the consistency and comparability of results across diverse settings.

Additionally, it is imperative to expand data collection efforts beyond the traditional clinical metrics. Gathering comprehensive information on aspects such as care delivery processes, health education initiatives, and patient attendance patterns can offer invaluable insights.

Furthermore, it is imperative to expand data collection efforts beyond the traditional clinical metrics. Gathering comprehensive information on aspects such as care delivery processes, health education initiatives, and patient attendance patterns can offer invaluable insights. These insights not only aid in shaping health policy planning but also provide a holistic perspective on the multifaceted nature of hypertension management.

As we advance in our understanding of hypertension care cascades, it is essential that future research places a strong emphasis on the incorporation of quality-adjusted service metrics. These metrics serve as vital tools in driving the development of successful hypertension management systems. They enable us to assess not only the quantity but also the quality of healthcare services, ultimately leading to more effective and patient-centered care.

## Supplementary Material

Supplementary Data sheet 1 JBI Checklist

Supplementary Data Sheet 2 Supplementary Material S1

Supplementary Data sheet 3 Supplementary Material S2

Supplementary Data sheet 4 Supplementary Material S3

Supplementary Data sheet 5 Supplementary Material S4

Supplementary Data sheet 6 Supplementary Material S5

Supplementary Data sheet 7 Supplementary Figures (S1-S38)
