# Supplementary material for: Care Cascades for Hypertension in Low-Income Settings: A Systematic Review and Meta-Analysis
Source: Int J Public Health. 2023 Oct 12;68:1606428. doi: 10.3389/ijph.2023.1606428 (PMC10600349; doi:10.3389/ijph.2023.1606428)
Supplement: Supplementary file 4 [file DataSheet3.docx]

| PubMed 30/04/2023 | ((cascade[tiab] OR continuum[tiab] OR linkage to care[tiab] OR retention in care[tiab] OR treatment access[tiab] OR engagement[tiab] OR awareness[tiab]) AND ((Hypertension[Mesh] OR hypertension[tiab] OR "Blood Pressure"[tiab] OR "High Blood Pressure*"[tiab]) NOT ("Hypertension, Pregnancy-Induced"[Mesh] OR "pregnancy"[tiab])) AND ("Africa"[Mesh] OR africa[tiab] OR maghreb*[tiab] OR maghrib*[tiab] OR Sahara*[tiab] OR sub-saharan*[tiab] OR "Afghanistan"[tiab] OR "Algeria"[tiab] OR "Angola"[tiab] OR "India"[tiab] OR "Samoa"[tiab] OR "Bangladesh"[tiab] OR "Belize"[tiab] OR "Bolivia"[tiab] OR "Benin"[tiab] OR "Botswana"[tiab] OR "Burkina Faso"[tiab] OR Burundi[tiab] OR "British Indian Ocean Territory"[tiab] OR "Bhutan"[tiab] OR "Cape Verde"[tiab] OR "Cambodia"[tiab] OR "Cameroon"[tiab] OR "Central African Republic"[tiab] OR "Ceuta"[tiab] OR Chad[tiab] OR Comoros[tiab] OR "Cote d'Ivoire"[tiab] OR "Egypt"[tiab] OR "El Salvador"[tiab] OR "Eritrea"[tiab] OR "Ethiopia"[tiab] OR "Equatorial Guinea"[tiab] OR "Eswatini"[tiab] OR "Gabon"[tiab] OR "Gambia"[tiab] OR "Ghana"[tiab] OR "Gaza"[tiab] OR "West Bank"[tiab] OR "Palestine"[tiab] OR "Guinea"[tiab] OR "Guinea-Bissau"[tiab] OR "Djibouti"[tiab] OR "Haiti"[tiab] OR "Honduras "[tiab] OR "Indonesia"[tiab] OR "Islamic Republic of Iran"[tiab] OR "Iran"[tiab] OR "Ivory Coast"[tiab] OR "Kenya"[tiab] OR "Kiribati"[tiab] OR "Kyrgyz Republic"[tiab] OR "Kyrgyzstan"[tiab] OR "Lao PDR"[tiab] OR "Lesotho"[tiab] OR "Liberia"[tiab] OR "Libya"[tiab] OR "Madeira"[tiab] OR "Melilla"[tiab] OR Morocco[tiab] OR "Madagascar"[tiab] OR "Malawi"[tiab] OR "Mauritania"[tiab] OR "Mauritius"[tiab] OR "Mayotte"[tiab] OR "Mongolia"[tiab] OR "Mozambique"[tiab] OR "Mali"[tiab] OR Namibia[tiab] OR "Nepal"[tiab] OR "Niger"[tiab] OR "Nigeria"[tiab] OR "Nicaragua"[tiab] OR "North Korea"[tiab] OR "Korea, Democratic People's Republic of"[tiab] OR "Democratic People's Republic of Korea "[tiab] OR "Papua New Guinea"[tiab] OR "Philippines"[tiab] OR "Reunion"[tiab] OR "Rwanda"[tiab] OR "Republic of the Congo"[tiab] OR "Democratic Republic of the Congo"[tiab] OR "Congo"[tiab] OR "Micronesia"[tiab] OR "Myanmar"[tiab] OR "Saint Helena"[tiab] OR "São Tomé and Principe"[tiab] OR "Solomon Islands"[tiab] OR "Pakistan"[tiab] OR "Seychelles"[tiab] OR "Sierra Leone"[tiab] OR "South Africa"[tiab] OR "Somalia"[tiab] OR "Senegal"[tiab] OR "Sri Lanka"[tiab] OR "Syria"[tiab] OR "Sudan"[tiab] OR "South Sudan"[tiab] OR "Swaziland"[tiab] OR "Sao Tome and Principe"[tiab] OR "Tajikistan"[tiab] OR "Tanzania"[tiab] OR "Timor-Leste"[tiab] OR "Togo"[tiab] OR "Tunisia"[tiab] OR "Uganda"[tiab] OR "Ukraine"[tiab] OR "Uzbekistan"[tiab] OR "Vanuatu"[tiab] OR "Vietnam"[tiab] OR "Yemen"[tiab] OR "Western Sahara"[tiab] OR "Zambia"[tiab] OR "Zimbabwe"[tiab])) | 1021 |
| --- | --- | --- |
| Embase  30/04/2023 | Combined with boolean term “AND” and “NOT”  #1 AND #2 AND #3  #1  ('care cascade':ti,ab OR 'treatment access':ti,ab OR 'awareness':ti,ab) AND [2010-2023]/py  #2  ('hypertension') AND [2010-2023]/py  #3  ('afghanistan' OR 'algeria' OR 'india' OR 'samoa' OR 'angola' OR 'bangladesh' OR 'belize' OR 'bolivia' OR 'benin' OR 'botswana' OR 'burkina faso' OR 'burundi' OR 'british indian ocean territory' OR 'bhutan' OR 'cape verde' OR 'cambodia' OR 'cameroon' OR 'central african republic' OR 'chad' OR 'comoros' OR 'cote d ivoire' OR 'egypt' OR 'el salvador' OR 'eritrea' OR 'eswatini' OR 'ethiopia' OR 'equatorial guinea' OR 'gabon' OR 'gambia' OR 'ghana' OR 'gaza' OR 'west bank' OR 'palestine' OR 'guinea' OR 'guinea-bissau' OR 'djibouti' OR 'haiti' OR 'honduras' OR 'indonesia' OR 'islamic republic of iran' OR 'iran' OR 'ivory coast' OR 'kenya' OR 'kiribati' OR 'kyrgyz republic' OR 'lao pdr' OR 'lesotho' OR 'liberia' OR 'libya' OR 'madeira' OR 'melilla' OR 'morocco' OR 'madagascar' OR 'malawi' OR 'mauritius' OR 'mayotte' OR 'mongolia' OR 'mozambique' OR 'mali' OR 'mauritania' OR 'namibia' OR 'nepal' OR 'niger' OR 'nigeria' OR 'nicaragua' OR 'north korea' OR 'democratic republic of korea' OR 'papua new guinea' OR 'philippines' OR 'reunion' OR 'rwanda' OR 'republic of the congo' OR 'democratic republic of the congo' OR 'congo' OR 'micronesia' OR 'myanmar' OR 'saint helena' OR 'são tomé' OR 'principe' OR 'solomon islands'/exp OR 'solomon islands' OR 'pakistan'/exp OR 'pakistan' OR 'seychelles'/exp OR 'seychelles' OR 'sierra leone' OR 'south africa' OR 'somalia' OR 'senegal' OR 'sri lanka' OR 'syria' OR 'sudan' OR 'south sudan' OR 'swaziland' OR 'sao tome' OR 'tajikistan' OR 'tanzania' OR 'timor-leste' OR 'togo' OR 'tunisia' OR 'uganda' OR 'ukraine' OR 'uzbekistan' OR 'vanuatu' OR 'vietnam' OR 'yemen' OR 'western sahara' OR 'zambia' OR 'zimbabwe') AND [2010-2022]/py  NOT ('pregnant' OR 'pregnancy' OR 'eclampsia' OR 'pre-eclampsia') AND [2010-2023]/py | 2279 |
| Scopus  30/04/2023 | TITLE-ABS ( ( “cascade” ) OR ( “continuum” ) OR ( “linkage to care” ) OR ( “retention in care” ) OR ( “treatment access” ) ) AND TITLE-ABS ( ( hypertension ) OR ( "High Blood Pressure*" ) ) AND TITLE-ABS ( ( africa ) OR ( maghreb* ) OR ( maghrib* ) OR ( sahara* ) OR ( sub-saharan* ) OR ( “afghanistan” ) OR ( “algeria” ) OR ( “india” ) OR ( “samoa” ) OR ( “Angola” ) OR ( “bangladesh” ) OR ( “belize” ) OR ( “bolivia” ) OR ( “benin” ) OR ( “botswana” ) OR ( "Burkina Faso" ) OR ( “burundi” ) OR ( "British Indian Ocean Territory" ) OR ( “bhutan” ) OR ( "Cape Verde" ) OR ( “cambodia” ) OR ( “cameroon” ) OR ( “Central African Republic” ) OR ( “ceuta” ) OR ( “chad” ) OR ( “comoros” ) OR ( “Cote d'Ivoire” ) OR ( “egypt” ) OR ( "El Salvador" ) OR ( “eritrea” ) OR ( “ethiopia” ) OR ( "Equatorial Guinea" ) OR ( “Eswatini” ) OR ( “gabon” ) OR ( “gambia” ) OR ( ghana ) OR ( "gaza" ) OR ( "West Bank" ) OR ( “palestine” ) OR ( “guinea” ) OR ( “guinea-bissau” ) OR ( djibouti ) OR ( "haiti" ) OR (“Honduras”) OR ( “indonesia” ) OR ( "Islamic Republic of Iran" ) OR ( "Iran") OR ( "Ivory Coast" ) OR ( "kenya" ) OR ( “kiribati” ) OR ( "Kyrgyz Republic" ) OR ( "Lao PDR" ) OR ( “lesotho” ) OR ( “liberia” ) OR ( “libya” ) OR ( “madeira” ) OR ( “melilla” ) OR ( “morocco” ) OR ( “madagascar” ) OR ( “malawi” ) OR ( “mauritania” ) OR ( “mauritius” ) OR ( “mayotte” ) OR ( “mongolia” ) OR ( “mozambique” ) OR ( “mali” ) OR ( “namibia” ) OR ( “nepal” ) OR ( “niger” ) OR ( “nigeria” ) OR ( “nicaragua” ) OR ( "North Korea" ) OR ( "Korea, Democratic People's Republic of" ) OR ( "Democratic People's Republic of Korea" ) OR ( "Papua New Guinea" ) OR ( “philippines” ) OR ( reunion ) OR ( rwanda ) OR ( "Republic of the Congo" ) OR ( "Democratic Republic of the Congo" ) OR ( “congo” ) OR ( myanmar ) OR ( "Saint Helena" ) OR ( "São Tomé and Principe" ) OR ( "Solomon Islands" ) OR ( “pakistan” ) OR ( seychelles ) OR ( "Sierra Leone" ) OR ( "South Africa" ) OR ( “somalia” ) OR ( “senegal” ) OR ( "Sri Lanka" ) OR ( “syria” ) OR ( “sudan” ) OR ( "South Sudan" ) OR ( “swaziland” ) OR ( "Sao Tome" ) OR ( “tajikistan” ) OR ( “tanzania” ) OR ( “timor-leste” ) OR ( “togo” ) OR ( “tunisia” ) OR ( “uganda” ) OR ( “ukraine” ) OR ( “uzbekistan” ) OR ( “vanuatu” ) OR ( “vietnam” ) OR ( “yemen” ) OR ( “Western Sahara” ) OR ( “zambia” ) OR ( “zimbabwe” ) ) AND ( LIMIT-TO ( PUBYEAR , 2023 ) OR ( LIMIT-TO ( PUBYEAR , 2022 ) OR LIMIT-TO ( PUBYEAR , 2021 ) OR LIMIT-TO ( PUBYEAR , 2020 ) OR LIMIT-TO ( PUBYEAR , 2019 ) OR LIMIT-TO ( PUBYEAR , 2018 ) OR LIMIT-TO ( PUBYEAR , 2017 ) OR LIMIT-TO ( PUBYEAR , 2016 ) OR LIMIT-TO ( PUBYEAR , 2015 ) OR LIMIT-TO ( PUBYEAR , 2014 ) OR LIMIT-TO ( PUBYEAR , 2013 ) OR LIMIT-TO ( PUBYEAR , 2012 ) OR LIMIT-TO ( PUBYEAR , 2011 ) OR LIMIT-TO ( PUBYEAR , 2010 ) ) | 95 |
| CINHAL 30/04/2023 | care cascade AND ( hypertension or high blood pressure ) | 16 |
