# Supplementary material for: Care Cascades for Hypertension in Low-Income Settings: A Systematic Review and Meta-Analysis
Source: Int J Public Health. 2023 Oct 12;68:1606428. doi: 10.3389/ijph.2023.1606428 (PMC10600349; doi:10.3389/ijph.2023.1606428)
Supplement: Supplementary file 5 [file DataSheet5.docx]

| ID | Q1 | Q2 | Q3 | Q4 | Q5 | Q6 | Q7 | Q8 | Q9 | JBI | Risk_of_Bias |
| --- | --- | --- | --- | --- | --- | --- | --- | --- | --- | --- | --- |
| 1 | Yes | Yes | Unclear | Yes | Unclear | Yes | Yes | Yes | Yes | 7 | Low |
| 2 | No | No | Yes | No | Unclear | Yes | Yes | Unclear | Unclear | 3 | High |
| 3 | No | No | Yes | No | Unclear | Yes | Yes | Unclear | Unclear | 3 | High |
| 4 | Yes | Yes | Yes | Unclear | Yes | Unclear | No | No | Yes | 5 | Moderate |
| 5 | No | No | Yes | No | Unclear | Yes | Yes | Unclear | Unclear | 3 | High |
| 6 | No | No | Yes | No | Unclear | Yes | Yes | Unclear | Unclear | 3 | High |
| 7 | Unclear | No | Unclear | No | Unclear | Yes | Yes | No | Unclear | 2 | High |
| 8 | Unclear | No | Unclear | Yes | Yes | Yes | Unclear | No | Unclear | 3 | High |
| 9 | Yes | Yes | No | Yes | Yes | Yes | Yes | No | Unclear | 6 | Moderate |
| 10 | Unclear | Yes | Yes | No | Yes | Yes | Yes | Unclear | Unclear | 5 | Moderate |
| 11 | No | No | Yes | No | Unclear | Yes | Yes | No | Unclear | 3 | High |
| 12 | No | No | Yes | No | Unclear | Yes | Yes | No | Unclear | 3 | High |
| 13 | Yes | Yes | Yes | Unclear | Yes | Yes | Yes | Yes | Yes | 8 | Low |
| 14 | No | Yes | Yes | Yes | Yes | Yes | Yes | Yes | Unclear | 7 | Low |
| 15 | Yes | Yes | Yes | Yes | Yes | Yes | Yes | Yes | Yes | 9 | Low |
| 16 | Yes | Yes | Unclear | Yes | Unclear | Yes | Yes | Unclear | Unclear | 5 | Moderate |
| 17 | No | No | Unclear | Yes | Unclear | Yes | Yes | Yes | Unclear | 4 | High |
| 18 | No | No | Unclear | Yes | Unclear | Yes | Yes | Yes | Unclear | 4 | High |
| 19 | No | No | Unclear | No | Unclear | Yes | Yes | Yes | Unclear | 3 | High |
| 20 | Unclear | No | Unclear | Unclear | Unclear | Yes | Yes | No | Unclear | 2 | High |
| 21 | Yes | Unclear | Yes | Yes | Unclear | Yes | Yes | Yes | Yes | 7 | Low |
| 22 | Unclear | Unclear | Unclear | Yes | Unclear | Yes | Yes | Yes | Unclear | 4 | High |
| 23 | Yes | Yes | Yes | Yes | Yes | Yes | Yes | Yes | Yes | 9 | Low |
| 24 | No | No | Unclear | Yes | Unclear | Yes | Yes | Yes | Unclear | 4 | High |
| 25 | No | No | Unclear | Yes | Unclear | Yes | Yes | Yes | Unclear | 4 | High |
| 26 | Unclear | Yes | Unclear | No | Yes | Yes | Yes | No | Yes | 5 | Moderate |
| 27 | Unclear | No | No | Yes | Unclear | Yes | Yes | Yes | Unclear | 4 | High |
| 28 | Yes | Yes | Unclear | Yes | Unclear | Yes | Yes | Yes | Yes | 7 | Low |
| 29 | Yes | Yes | Yes | Yes | Yes | Unclear | Unclear | Yes | Yes | 7 | Low |
| 30 | No | Yes | No | Yes | Unclear | Yes | Yes | Yes | Yes | 6 | Moderate |
| 31 | Yes | Yes | Yes | Yes | Yes | Yes | Unclear | Yes | Yes | 8 | Low |
| 32 | Yes | Yes | Unclear | Yes | Yes | Yes | Yes | Yes | Yes | 8 | Low |
| 33 | Yes | Yes | Unclear | Yes | Unclear | Yes | Yes | Yes | Unclear | 6 | Moderate |
| 34 | Yes | Yes | Yes | Yes | Yes | Yes | Unclear | Yes | Yes | 8 | Low |
| 35 | Yes | Yes | Yes | Yes | Unclear | Unclear | Unclear | Yes | Yes | 6 | Moderate |
| 36 | Yes | Yes | Unclear | Yes | Unclear | Yes | Yes | Yes | Unclear | 6 | Moderate |
| 37 | Yes | Yes | Yes | Yes | Yes | Yes | Unclear | Yes | Yes | 8 | Low |
| 38 | Yes | Yes | Yes | Yes | Yes | Yes | Unclear | Yes | Yes | 8 | Low |
| 39 | Yes | Yes | Unclear | Yes | Yes | Yes | Yes | Yes | Unclear | 7 | Low |
| 40 | Yes | Yes | Yes | Yes | Unclear | Yes | Yes | Yes | Unclear | 7 | Low |
| 41 | Unclear | Yes | Yes | Yes | Unclear | Yes | Unclear | Yes | Unclear | 5 | Moderate |
| 42 | Yes | Yes | Yes | Yes | Unclear | Yes | Yes | Yes | Yes | 8 | Low |
| 43 | No | No | Unclear | Unclear | Unclear | Yes | Yes | Yes | Unclear | 3 | High |
| 44 | Yes | Unclear | Unclear | Yes | Unclear | Yes | Yes | Yes | Unclear | 5 | Moderate |
| 45 | Yes | Yes | Yes | Yes | Unclear | Yes | Yes | Yes | Unclear | 7 | Low |
| 46 | No | No | Unclear | Yes | Unclear | Yes | Yes | Yes | Unclear | 4 | High |
| 47 | Yes | Yes | Yes | Unclear | Unclear | Yes | Yes | Unclear | Yes | 6 | Moderate |
| 48 | Yes | Unclear | Yes | Unclear | Unclear | Unclear | Unclear | Unclear | Unclear | 2 | High |
| 49 | Yes | Yes | Yes | Yes | Unclear | Yes | Yes | Yes | Unclear | 7 | Low |
| 50 | Yes | Yes | Yes | Yes | Unclear | Yes | Yes | Yes | Unclear | 7 | Low |
| 51 | No | Yes | Yes | Yes | Unclear | Unclear | Unclear | Yes | Unclear | 4 | High |
| 52 | Yes | Yes | Yes | No | No | Yes | No | No | Unclear | 4 | High |
| 53 | Yes | Yes | Yes | No | No | Yes | No | No | Unclear | 4 | High |
| 54 | Yes | Yes | Yes | No | No | Yes | No | No | Unclear | 4 | High |
| 55 | Yes | Yes | Unclear | Yes | Unclear | Yes | Yes | Yes | Unclear | 6 | Moderate |
| 56 | Yes | Yes | Yes | Yes | Yes | Yes | Yes | Yes | Yes | 9 | Low |
| 57 | Yes | Yes | Yes | Yes | Yes | Yes | Yes | Yes | Yes | 9 | Low |
| 58 | Unclear | Yes | No | Yes | Yes | Yes | Yes | Yes | Yes | 7 | Low |
| 59 | Unclear | Yes | Yes | Yes | Yes | Yes | Yes | Yes | Yes | 8 | Low |
| 60 | Yes | Yes | Yes | Yes | Unclear | Yes | Yes | Yes | Unclear | 7 | Low |
| 61 | Unclear | No | Unclear | Yes | No | Yes | Yes | Unclear | Unclear | 3 | High |
| 62 | Yes | Yes | Yes | Yes | Yes | Yes | Unclear | No | Yes | 7 | Low |
| 63 | No | No | Yes | Unclear | Unclear | Yes | Yes | Yes | Unclear | 4 | High |
| 64 | No | No | Yes | Unclear | Unclear | Yes | Yes | Yes | Unclear | 4 | High |
| 65 | Yes | Yes | Yes | Yes | Yes | Yes | Yes | Yes | Yes | 9 | Low |
| 66 | Yes | Yes | Yes | Yes | Yes | Yes | Yes | Yes | Yes | 9 | Low |
| 67 | Yes | Yes | Yes | Yes | Yes | Yes | Yes | Yes | Yes | 9 | Low |
| 68 | Yes | Yes | Yes | Yes | Yes | Yes | Yes | Yes | Yes | 9 | Low |
| 69 | Yes | Yes | Yes | Yes | Yes | Yes | Yes | Yes | Yes | 9 | Low |
| 70 | No | Yes | Yes | Yes | Unclear | Yes | Yes | Yes | Yes | 7 | Low |
| 71 | Yes | Unclear | No | Yes | Yes | Yes | Yes | Yes | Unclear | 6 | Moderate |
| 72 | Yes | Yes | Yes | Yes | No | Yes | Yes | Yes | Unclear | 7 | Low |
| 73 | No | No | Yes | Unclear | Unclear | No | Unclear | Unclear | Unclear | 1 | High |
| 74 | No | No | Yes | Unclear | Unclear | No | Unclear | Unclear | Unclear | 1 | High |
| 75 | Yes | Yes | Yes | Yes | Yes | Yes | Yes | Yes | Yes | 9 | Low |
| 76 | No | No | Yes | Unclear | Unclear | Unclear | Unclear | No | Unclear | 1 | High |
| 77 | Unclear | No | Unclear | Unclear | Unclear | Unclear | Yes | Yes | Unclear | 2 | High |
| 78 | Yes | Yes | Yes | No | Unclear | Yes | Yes | Unclear | Unclear | 5 | Moderate |
| 79 | No | No | Yes | Yes | Yes | Yes | Yes | Yes | Unclear | 6 | Moderate |
| 80 | Unclear | No | Yes | Unclear | Unclear | Yes | Yes | No | Unclear | 3 | High |
| 81 | No | No | Yes | No | Unclear | Unclear | Unclear | Yes | Unclear | 2 | High |
| 82 | Unclear | No | Yes | Yes | Unclear | Unclear | Yes | Unclear | Unclear | 3 | High |
| 83 | Unclear | Unclear | Unclear | Yes | Yes | Yes | Unclear | Yes | Yes | 5 | Moderate |
| 84 | Yes | Yes | Unclear | Yes | Unclear | Yes | Yes | Unclear | Unclear | 5 | Moderate |
| 85 | Yes | Yes | Unclear | Yes | Unclear | Yes | Unclear | Unclear | Unclear | 4 | High |
| 86 | Unclear | Yes | Unclear | Yes | Yes | Yes | Yes | Yes | Yes | 7 | Low |
| 87 | No | No | Yes | Unclear | Unclear | Yes | Unclear | Yes | Unclear | 3 | High |
| 88 | No | No | No | Yes | Yes | Yes | Unclear | Yes | Unclear | 4 | High |
| 89 | Yes | Yes | Yes | Unclear | Yes | Yes | Yes | Yes | Yes | 8 | Low |
| 90 | Yes | Yes | Yes | Unclear | Yes | Yes | Yes | Yes | Yes | 8 | Low |
| 91 | Yes | Yes | Yes | Yes | Yes | Yes | Yes | Yes | Unclear | 8 | Low |
| 92 | No | No | Yes | Yes | Yes | Yes | Yes | No | Yes | 6 | Moderate |
| 93 | No | No | Yes | Unclear | Unclear | Yes | Yes | Yes | Unclear | 4 | High |
| 94 | Yes | Yes | Yes | Yes | Yes | Yes | Yes | Yes | Yes | 9 | Low |
| 95 | Yes | Yes | Yes | Yes | Yes | Yes | No | Yes | Unclear | 7 | Low |
| 96 | No | Yes | Unclear | Yes | Unclear | Yes | Yes | Yes | Unclear | 5 | Moderate |
| 97 | No | No | Yes | No | Unclear | Yes | Yes | No | Unclear | 3 | High |
| 98 | Unclear | No | Yes | No | Unclear | Yes | Yes | Unclear | Unclear | 3 | High |
| 98 | Yes | Yes | Yes | Yes | Yes | Yes | Yes | Yes | Yes | 9 | Low |
| 100 | No | No | Yes | Unclear | Unclear | Yes | Yes | Yes | Unclear | 4 | High |

***A JBI score <5 was associated to a high risk of bias, 5-6 to a moderate and 7-9 to a low one.***

***References***

1 Pires JE, Sebastião YV, Langa AJ, Nery SV. Hypertension in Northern Angola: prevalence, associated factors, awareness, treatment and control. BMC Public Health 2013;13:90. https://doi.org/10.1186/1471-2458-13-90.

2 Victória Pereira S, Neto M, Feijão A, Oliveira P, Brandão M, Soito E, et al. May Measurement Month 2018: an analysis of blood pressure screening results from Angola. Eur Heart J Suppl 2020;22:H8–10. https://doi.org/10.1093/eurheartj/suaa015.

3 Victória Pereira S, Neto M, Feijão A, Lutucuta E, Mbala C, Muela H, et al. May Measurement Month 2019: an analysis of blood pressure screening results from Angola. Eur Heart J Suppl 2021;23:B9–11. https://doi.org/10.1093/eurheartj/suab038.

4 Rahman MdM, Gilmour S, Akter S, Abe SK, Saito E, Shibuya K. Prevalence and control of hypertension in Bangladesh: a multilevel analysis of a nationwide population-based survey. J Hypertens 2015;33:465–72. https://doi.org/10.1097/HJH.0000000000000421.

5 Malik F-T-N, Al Mamun MA, Ishraquzzaman M, Kalimuddin M, Shahriar Huq T, Rahman MS, et al. May Measurement Month 2018: an analysis of blood pressure screening results from Bangladesh. Eur Heart J Suppl 2020;22:H20–2. https://doi.org/10.1093/eurheartj/suaa086.

6 Malik F-T-N, Al Mamun MA, Choudhury SR, Ishraquzzaman M, Kalimuddin M, Huq TS, et al. May Measurement Month 2019: an analysis of blood pressure screening results from Bangladesh. Eur Heart J Suppl 2021;23:B21–3. https://doi.org/10.1093/eurheartj/suab017.

7 Houehanou C, Sonou A, Adjagba P, Dohou H, Hounkponou M, Kpolédji G, et al. May Measurement Month 2018: an analysis of blood pressure screening results from Benin. Eur Heart J Suppl 2022;24:F9–11. https://doi.org/10.1093/eurheartjsupp/suac039.

8 Desormais I, Amidou SA, Houehanou YC, Houinato SD, Gbagouidi GN, Preux PM, et al. The prevalence, awareness, management and control of hypertension in men and women in Benin, West Africa: the TAHES study. BMC Cardiovasc Disord 2019;19:303. https://doi.org/10.1186/s12872-019-01273-7.

9 Doulougou B, Kouanda S, Ouédraogo GH, Meda BI, Bado A, Zunzunegui MV. Awareness, treatment, control of hypertension and utilization of health care services following screening in the North-central region of Burkina Faso. Pan Afr Med J 2014;19. https://doi.org/10.11604/pamj.2014.19.259.4707.

10 Cissé K, Kouanda S, Coppieters’t Wallant Y, Kirakoya-Samadoulougou F. Awareness, Treatment, and Control of Hypertension among the Adult Population in Burkina Faso: Evidence from a Nationwide Population-Based Survey. Int J Hypertens 2021;2021:1–9. https://doi.org/10.1155/2021/5547661.

11 Azevedo V, Dias L, Soares I, Garcia G, Xia X, Ster AC, et al. May Measurement Month 2018: an analyses of blood pressure screening results from Cabo Verde. Eur Heart J Suppl 2020;22:H30–2. https://doi.org/10.1093/eurheartj/suaa020.

12 Azevedo V, Dias L, Garcia G, Soares I, Silva M, Delgado I, et al. May Measurement Month 2019: an analysis of blood pressure screening results from Cape Verde. Eur Heart J Suppl 2021;23:B37–9. https://doi.org/10.1093/eurheartj/suab053.

13 Chham S, Buffel V, Van Olmen J, Chhim S, Ir P, Wouters E. The cascade of hypertension care in Cambodia: evidence from a cross-sectional population-based survey. BMC Health Serv Res 2022;22:838. https://doi.org/10.1186/s12913-022-08232-7.

14 Tianyi FL, Agbor VN, Njamnshi AK. Prevalence, awareness, treatment, and control of hypertension in Cameroonians aged 50 years and older: A community-based study. Health Sci Rep 2018;1:e44. https://doi.org/10.1002/hsr2.44.

15 Lemogoum D, Van de Borne P, Lele CEB, Damasceno A, Ngatchou W, Amta P, et al. Prevalence, awareness, treatment, and control of hypertension among rural and urban dwellers of the Far North Region of Cameroon. J Hypertens 2018;36:159–68. https://doi.org/10.1097/HJH.0000000000001513.

16 Mbouemboue OP, Ngoufack TJO. High Blood Pressure Prevalence, Awareness, Control, and Associated Factors in a Low-Resource African Setting. Front Cardiovasc Med 2019;6:119. https://doi.org/10.3389/fcvm.2019.00119.

17 Dzudie A, Njume E, Boombhi J, Awungia A, Ndom MS, Ebasone PV, et al. May Measurement Month 2018: blood pressure screening results in Cameroon. Eur Heart J Suppl 2020;22:H33–6. https://doi.org/10.1093/eurheartj/suaa019.

18 Dzudie A, Njume E, Mfekeu LK, Djomou A, Ba H, Ndom MS, et al. May Measurement Month 2019: an analysis of blood pressure screening results from Cameroon. Eur Heart J Suppl 2021;23:B33–6. https://doi.org/10.1093/eurheartj/suab056.

19 Ellenga Mbolla BF, Kouala Landa CM, Bakekolo PR, Makani Bassakouahou JK, Bouithy SN, Eyeni-Sinomono T, et al. May Measurement Month 2018: an analysis of blood pressure screening results from Republic of the Congo. Eur Heart J Suppl 2020;22:H47–9. https://doi.org/10.1093/eurheartj/suaa026.

20 Ellenga-Mbolla B, Makani-Bassakouahou J, Landa CK, Monabeka M-G, Ossou-Nguiet P-M, Ngamami SM, et al. May Measurement Month 2019: an analysis of blood pressure screening results from Republic of the Congo. Eur Heart J Suppl 2021;23:B49–51. https://doi.org/10.1093/eurheartj/suab028.

21 Katchunga PB, M’Buyamba-Kayamba J-R, Masumbuko BE, Lemogoum D, Kashongwe ZM, Degaute J-P, et al. Hypertension artérielle chez l’adulte Congolais du Sud Kivu : résultats de l’étude Vitaraa. Presse Médicale 2011;40:e315–23. https://doi.org/10.1016/j.lpm.2010.10.036.

22 Buila NB, Ngoyi GN, Bayauli PM, Katamba FK, Lubenga YN, Kazadi SM, et al. Analysis of blood pressure and selected cardiovascular risk factors in the Democratic Republic of the Congo: the May Measurement Month 2018 results. Eur Heart J Suppl 2020;22:H50–2. https://doi.org/10.1093/eurheartj/suaa027.

23 Sanuade OA, Awuah RB, Kushitor M. Hypertension awareness, treatment and control in Ghana: a cross-sectional study. Ethn Health 2020;25:702–16. https://doi.org/10.1080/13557858.2018.1439898.

24 Twumasi-Ankrah B, Myers-Hansen GA, Adu-Boakye Y, Tannor EK, Nyarko OO, Boakye E, et al. May Measurement Month 2018: an analysis of blood pressure screening results from Ghana. Eur Heart J Suppl 2020;22:H59–61. https://doi.org/10.1093/eurheartj/suaa029.

25 Twumasi-Ankrah B, Poulter NR, Tannor EK, Adu-Boakye Y, Nyarko OO, Opoku G, et al. May Measurement Month 2019: an analysis of blood pressure screening results from Ghana-Sub-Saharan Africa. Eur Heart J Suppl 2021;23:B62–5. https://doi.org/10.1093/eurheartj/suab023.

26 Turé R, Damasceno A, Djicó M, Lunet N. Prevalence, awareness, treatment, and control of hypertension in Bissau, Western Africa. J Clin Hypertens 2022;24:358–61. https://doi.org/10.1111/jch.14443.

27 Polsinelli VB, Satchidanand N, Singh R, Holmes D, Izzo JL. Hypertension and aging in rural Haiti: results from a preliminary survey. J Hum Hypertens 2017;31:138–44. https://doi.org/10.1038/jhh.2016.52.

28 Metz M, Pierre JL, Yan LD, Rouzier V, St‐Preux S, Exantus S, et al. Hypertension continuum of care: Blood pressure screening, diagnosis, treatment, and control in a population‐based cohort in Haiti. J Clin Hypertens 2022;24:246–54. https://doi.org/10.1111/jch.14399.

29 Kaur P, Rao SR, Radhakrishnan E, Rajasekar D, Gupte MD. Prevalence, awareness, treatment, control and risk factors for hypertension in a rural population in South India. Int J Public Health 2012;57:87–94. https://doi.org/10.1007/s00038-011-0303-3.

30 Yip W, Wong TY, Jonas JB, Zheng Y, Lamoureux EL, Nangia V, et al. Prevalence, awareness, and control of hypertension among Asian Indians living in urban Singapore and rural India. J Hypertens 2013;31:1539–46. https://doi.org/10.1097/HJH.0b013e328361d52b.

31 Geevar Z, Krishnan MN, Venugopal K, Sanjay G, Harikrishnan S, Mohanan PP, et al. Prevalence, Awareness, Treatment, and Control of Hypertension in Young Adults (20–39 Years) in Kerala, South India. Front Cardiovasc Med 2022;9:765442. https://doi.org/10.3389/fcvm.2022.765442.

32 Banerjee S, Mukherjee TK, Basu S. Prevalence, awareness, and control of hypertension in the slums of Kolkata. Indian Heart J 2016;68:286–94. https://doi.org/10.1016/j.ihj.2015.09.029.

33 Cao Y, Sathish T, Haregu T, Wen Y, Mello GT de, Kapoor N, et al. Factors Associated With Hypertension Awareness, Treatment, and Control Among Adults in Kerala, India. Front Public Health 2021;9:753070. https://doi.org/10.3389/fpubh.2021.753070.

34 Janki B, Mohan Singh RC, Sadhana A. Prevalence, Awareness, Treatment and Control of Hypertension among the Elderly Residing in Rural Area of Haldwani Block, in Nainital District of Uttarakhand. J Cardiovasc Dis Res 2016;7:112–5. https://doi.org/10.5530/jcdr.2016.3.3.

35 Karmakar N, Nag K, Saha I, Parthasarathi R, Patra M, Sinha R. Awareness, treatment, and control of hypertension among adult population in a rural community of Singur block, Hooghly District, West Bengal. J Educ Health Promot 2018;7:134. https://doi.org/10.4103/jehp.jehp_164_18.

36 Negi PC, Chauhan R, Rana V, Vidyasagar, Lal K. Epidemiological study of non-communicable diseases (NCD) risk factors in tribal district of Kinnaur, HP: A cross-sectional study. Indian Heart J 2016;68:655–62. https://doi.org/10.1016/j.ihj.2016.03.002.

37, 38 Thakur JS, Nangia R. Prevalence, Awareness, Treatment, and Control of Hypertension and Diabetes: Results From Two State-Wide STEPS Survey in Punjab and Haryana, India. Front Public Health 2022;10:768471. https://doi.org/10.3389/fpubh.2022.768471.

39 Amarchand R, Kulothungan V, Krishnan A, Mathur P. Hypertension treatment cascade in India: results from National Noncommunicable Disease Monitoring Survey. J Hum Hypertens 2022. https://doi.org/10.1038/s41371-022-00692-y.

40 Boro B, Banerjee S. Decomposing the rural–urban gap in the prevalence of undiagnosed, untreated and under-treated hypertension among older adults in India. BMC Public Health 2022;22:1310. https://doi.org/10.1186/s12889-022-13664-1.

41 Kothavale A, Puri P, Sangani PG. Quantifying population level hypertension care cascades in India: a cross-sectional analysis of risk factors and disease linkages. BMC Geriatr 2022;22:98. https://doi.org/10.1186/s12877-022-02760-x.

42 Lee J, Wilkens J, Meijer E, Sekher TV, Bloom DE, Hu P. Hypertension awareness, treatment, and control and their association with healthcare access in the middle-aged and older Indian population: A nationwide cohort study. PLOS Med 2022;19:e1003855. https://doi.org/10.1371/journal.pmed.1003855.

43 Maheshwari A, Verma N, Bhardwaj S, Jose AP, Bhalla S, More A, et al. May Measurement Month 2018: an analysis of blood pressure screening campaign results in India. Eur Heart J Suppl 2020;22:H62–5. https://doi.org/10.1093/eurheartj/suaa030.

44 Saju MD, Allagh KP, Scaria L, Joseph S, Thiyagarajan JA. Prevalence, Awareness, Treatment, and Control of Hypertension and Its Associated Risk Factors: Results from Baseline Survey of SWADES Family Cohort Study. Int J Hypertens 2020;2020:1–7. https://doi.org/10.1155/2020/4964835.

45 Saxena V, Kalyani V, Kodi S M, Dhar M, Verma A, Senkadhirdasan, et al. Control of Blood Pressure in District Dehradun, India: is Rule of Halves Still Valid? Indian J Public Health Res Dev 2021. https://doi.org/10.37506/ijphrd.v12i3.16122.

46 Patil M, Jose AP, More A, Maheshwari A, Verma N, Shah R, et al. May Measurement Month 2019: an analysis of blood pressure screening results from India. Eur Heart J Suppl 2021;23:B73–6. https://doi.org/10.1093/eurheartj/suab047.

47 Maniyara K, Kodali PB, Thankappan KR. Prevalence, awareness, treatment, control and correlates of prevalence and control of hypertension among older adults in Kerala: A mixed methods study. Indian Heart J 2023;75:185–9. https://doi.org/10.1016/j.ihj.2023.03.004.

48 Prashanth HL, Chandrashekar S, Madhusudhana M. Hypertension in Young Adults - An Urban and Rural Comparative Study. Indian J Public Health Res Dev 2013;4:168. https://doi.org/10.5958/j.0976-5506.4.4.166.

49 Singh S, Shankar R, Singh GP. Prevalence and Associated Risk Factors of Hypertension: A Cross-Sectional Study in Urban Varanasi. Int J Hypertens 2017;2017:1–10. https://doi.org/10.1155/2017/5491838.

50 Geldsetzer P, Tan MM, Dewi F, Quyen B, Juvekar S, Hanifi S, et al. Hypertension care in demographic surveillance sites: a cross-sectional study in Bangladesh, India, Indonesia, Malaysia, Viet Nam. Bull World Health Organ 2022;100:601–9. https://doi.org/10.2471/BLT.22.287807.

51 Malekzadeh MM, Etemadi A, Kamangar F, Khademi H, Golozar A, Islami F, et al. Prevalence, awareness and risk factors of hypertension in a large cohort of Iranian adult population. J Hypertens 2013;31:1364–71. https://doi.org/10.1097/HJH.0b013e3283613053.

52, 53, 54 Esteghamati A, Etemad K, Koohpayehzadeh J, Abbasi M, Meysamie A, Khajeh E, et al. Awareness, Treatment and Control of Pre-hypertension and Hypertension among Adults in Iran. Arch Iran Med 2016;19:456–64.

55 Eghbali M, Khosravi A, Feizi A, Mansouri A, Mahaki B, Sarrafzadegan N. Prevalence, awareness, treatment, control, and risk factors of hypertension among adults: a cross-sectional study in Iran. Epidemiol Health 2018;40:e2018020. https://doi.org/10.4178/epih.e2018020.

56 Mirzaei M, Mirzaei M, Bagheri B, Dehghani A. Awareness, treatment, and control of hypertension and related factors in adult Iranian population. BMC Public Health 2020;20:667. https://doi.org/10.1186/s12889-020-08831-1.

57 Rajati F, Hamzeh B, Pasdar Y, Safari R, Moradinazar M, Shakiba E, et al. Prevalence, awareness, treatment, and control of hypertension and their determinants: Results from the first cohort of non-communicable diseases in a Kurdish settlement. Sci Rep 2019;9:12409. https://doi.org/10.1038/s41598-019-48232-y.

58 Nikparvar M, Farshidi H, Madani A, Rad RE, Azad M, Eftekhaari TE, et al. Prevalence, Awareness, Treatment, and Control of Hypertension in Hormozgan Province, Iran. Int Cardiovasc Res J 2019;13.

59 Oraii A, Shafiee A, Jalali A, Alaeddini F, Saadat S, Sadeghian S, et al. Prevalence, Awareness, Treatment, and Control of Hypertension among Adult Residents of Tehran: The Tehran Cohort Study. Glob Heart 2022;17:31. https://doi.org/10.5334/gh.1120.

60 Mohamed SF, Mutua MK, Wamai R, Wekesah F, Haregu T, Juma P, et al. Prevalence, awareness, treatment and control of hypertension and their determinants: results from a national survey in Kenya. BMC Public Health 2018;18:1219. https://doi.org/10.1186/s12889-018-6052-y.

61 Mirrakhimov E, Zakirov U, Abilova S, Asanbaev A, Bektasheva E, Asanaliev N, et al. May Measurement Month 2019: analysis of blood pressure screening in Bishkek, Kyrgyzstan. Eur Heart J Suppl 2022;24:F19–21. https://doi.org/10.1093/eurheartjsupp/suac042.

62 Pengpid S, Vonglokham M, Kounnavong S, Sychareun V, Peltzer K. The prevalence, awareness, treatment, and control of hypertension among adults: the first cross-sectional national population-based survey in Laos. Vasc Health Risk Manag 2019;Volume 15:27–33. https://doi.org/10.2147/VHRM.S199178.

63 Ndhlovu HLL, Masiye JK, Chirwa ML, Nyirenda NM, Dhlamini TD, Beaney T, et al. May Measurement Month 2018: an analysis of blood pressure screening results from Malawi. Eur Heart J Suppl 2020;22:H80–2. https://doi.org/10.1093/eurheartj/suaa034.

64 Ndhlovu HLL, Chirwa ML, Mbeba MK, Nyirenda NM, Mbulaje LD, Beaney T, et al. May Measurement Month 2019: an analysis of blood pressure screening results from Malawi. Eur Heart J Suppl 2021;23:B95–7. https://doi.org/10.1093/eurheartj/suab041.

65, 66, 68 Pengpid S, Peltzer K. National trends in prevalence, awareness, treatment, and control of hypertension among adults in Mongolia from 4 cross-sectional surveys in 2005, 2009, 2013, and 2019. Medicine (Baltimore) 2022;101:e30140. https://doi.org/10.1097/MD.0000000000030140.

67 Potts H, Baatarsuren U, Myanganbayar M, Purevdorj B, Lkhagvadorj B, Ganbat N, et al. Hypertension prevalence and control in Ulaanbaatar, Mongolia. J Clin Hypertens 2020;22:103–10. https://doi.org/10.1111/jch.13784.

69 Ko-Ko-Zaw, Tint-Swe-Latt, Phyu-Phyu-Aung, Thein-Gi-Thwin, Tin-Khine-Myint. Prevalence of Hypertension and Its Associated Factors in the Adult Population in Yangon Division, Myanmar. Asia Pac J Public Health 2011;23:496–506. https://doi.org/10.1177/1010539509349147.

70 Neupane D, Shrestha A, Mishra SR, Bloch J, Christensen B, McLachlan CS, et al. Awareness, Prevalence, Treatment, and Control of Hypertension in Western Nepal. Am J Hypertens 2017;30:907–13. https://doi.org/10.1093/ajh/hpx074.

71 Karmacharya BM, Koju RP, LoGerfo JP, Chan KCG, Mokdad AH, Shrestha A, et al. Awareness, treatment and control of hypertension in Nepal: findings from the Dhulikhel Heart Study. Heart Asia 2017;9:1–8. https://doi.org/10.1136/heartasia-2016-010766.

72 Khanal MK, Dhungana RR, Bhandari P, Gurung Y, Paudel KN. Prevalence, associated factors, awareness, treatment, and control of hypertension: Findings from a cross sectional study conducted as a part of a community based intervention trial in Surkhet, Mid-western region of Nepal. PLOS ONE 2017;12:e0185806. https://doi.org/10.1371/journal.pone.0185806.

73 Adhikari TB, Bhattarai H, Ranabhat K, Khanal P, Mishra SR, Koirala S, et al. May Measurement Month 2018: an analysis of blood pressure screening results from Nepal. Eur Heart J Suppl 2020;22:H92–5. https://doi.org/10.1093/eurheartj/suaa037.

74 Bhattarai H, McLachlan CS, Khanal P, Adhikari TB, Ranabhat K, Koirala S, et al. May Measurement Month 2019: an analysis of blood pressure screening results from Nepal. Eur Heart J Suppl 2021;23:B110–3. https://doi.org/10.1093/eurheartj/suab042.

75 Valladares MJ, Rodríguez Sándigo NA, Rizo Rivera GO, Rodríguez Jarquín MA, Rivera Castillo RM, López Bonilla IM. Prevalence, awareness, treatment, and control of hypertension in a small northern town in Nicaragua: The Elieth‐HIFARI study. Health Sci Rep 2019;2. https://doi.org/10.1002/hsr2.120.

76 Wahab KW, Kolo PM, Sani MU, Okubadejo NU, Peter JO, Aigbe F, et al. May Measurement Month 2018: an analysis of blood pressure screening results from Nigeria. Eur Heart J Suppl 2020;22:H96–9. https://doi.org/10.1093/eurheartj/suaa038.

77 Wahab KW, Kolo PM, Odili A, Iwuozo E, Ifebunandu N, Ademiluyi B, et al. May Measurement Month 2019: an analysis of blood pressure screening results from Nigeria. Eur Heart J Suppl 2021;23:B114–6. https://doi.org/10.1093/eurheartj/suab059.

78 Obagha CE, Danladi B, Kamateeka M, Chori BS, Ogbonnaya U, Maduka D, et al. Unmet needs of hypertension care in Nigeria: results of the community action against non-communicable diseases (COMAAND) project preintervention survey. Blood Press Monit 2022;27:27–32. https://doi.org/10.1097/MBP.0000000000000561.

79 Shafi ST, Shafi T. A survey of hypertension prevalence, awareness, treatment, and control in health screening camps of rural central Punjab, Pakistan. J Epidemiol Glob Health 2017;7:135. https://doi.org/10.1016/j.jegh.2017.01.001.

80 Memon FS, Wang W, Beaney T, Bai K, Poulter NR, Ishaq M. May Measurement Month 2018: an analysis of blood pressure screening results from Pakistan. Eur Heart J Suppl 2022;24:F31–3. https://doi.org/10.1093/eurheartjsupp/suac034.

80 Castillo RR, Mercado-Asis LB, Atilano AA, David-Ona DI, Diaz AF, Granada CN, et al. May Measurement Month 2018: an analysis of blood pressure screening in the Philippines. Eur Heart J Suppl 2020;22:H104–7. https://doi.org/10.1093/eurheartj/suaa040.

82 Diaz ABF, David-Ona DI, Mercado-Asis LB, Atilano AA, Vilela GC, Bonzon DD, et al. May Measurement Month 2019: an analysis of blood pressure screening results in the Philippines. Eur Heart J Suppl 2021;23:B120–3. https://doi.org/10.1093/eurheartj/suab061.

83 LaMonica LC, McGarvey ST, Rivara AC, Sweetman CA, Naseri T, Reupena MS, et al. Cascades of diabetes and hypertension care in Samoa: Identifying gaps in the diagnosis, treatment, and control continuum – a cross-sectional study. Lancet Reg Health - West Pac 2022;18:100313. https://doi.org/10.1016/j.lanwpc.2021.100313.

84 Duboz P, Boëtsch G, Gueye L, Macia E. Hypertension prevalence, awareness, treatment and control in Dakar (Senegal). J Hum Hypertens 2014;28:489–93. https://doi.org/10.1038/jhh.2013.142.

85 Duboz P, Boëtsch G, Gueye L, Macia E. Hypertension in the Ferlo (Northern Senegal): prevalence, awareness, treatment and control. Pan Afr Med J 2016;25:177.

86 Geraedts TJM, Boateng D, Lindenbergh KC, Delft D, Mathéron HM, Mönnink GLE, et al. Evaluating the cascade of care for hypertension in Sierra Leone. Trop Med Int Health 2021;26:1470–80. https://doi.org/10.1111/tmi.13664.

87 Beheiry HM, Abdalla AA, Fahal NA, Mohamed MI, Ibrahim DA, Medani SA, et al. May Measurement Month 2018: an analysis of blood pressure screening results from Sudan. Eur Heart J Suppl 2020;22:H122–4. https://doi.org/10.1093/eurheartj/suaa045.

88 Muhamedhussein MS, Nagri ZI, Manji KP. Prevalence, Risk Factors, Awareness, and Treatment and Control of Hypertension in Mafia Island, Tanzania. Int J Hypertens 2016;2016:1–5. https://doi.org/10.1155/2016/1281384.

89 Osetinsky B, Mhalu G, Mtenga S, Tediosi F. Care cascades for hypertension and diabetes: Cross-sectional evaluation of rural districts in Tanzania. PLoS Med 2022;19:1–17. https://doi.org/10.1371/journal.pmed.1004140.

90 Muhihi AJ, Anaeli A, Mpembeni RNM, Sunguya BF, Leyna G, Kakoko D, et al. Prevalence, Awareness, Treatment, and Control of Hypertension among Young and Middle-Aged Adults: Results from a Community-Based Survey in Rural Tanzania. Int J Hypertens 2020;2020:1–13. https://doi.org/10.1155/2020/9032476.

91 Ben Romdhane H, Ben Ali S, Skhiri H, Traissac P, Bougatef S, Maire B, et al. Hypertension among Tunisian adults: results of the TAHINA project. Hypertens Res 2012;35:341–7. https://doi.org/10.1038/hr.2011.198.

92 Boujnah R, Nazek L, Maalej M, Achhab YE, Nejjari C. Hypertension in Tunisian adults attending primary care physicians (ETHNA-Tunisia). Indian Heart J 2018;70:544–7. https://doi.org/10.1016/j.ihj.2017.11.005.

93 Haj Amor S, Beaney T, Saidi O, Clarke J, Poulter NR, Ben Alaya N, et al. May Measurement Month 2019: an analysis of blood pressure screening results from Tunisia. Eur Heart J Suppl 2021;23:B144–6. https://doi.org/10.1093/eurheartj/suab032.

94 Son PT, Quang NN, Viet NL, Khai PG, Wall S, Weinehall L, et al. Prevalence, awareness, treatment and control of hypertension in Vietnam—results from a national survey. J Hum Hypertens 2012;26:268–80. https://doi.org/10.1038/jhh.2011.18.

95 Ha DA, Goldberg RJ, Allison JJ, Chu TH, Nguyen HL. Prevalence, Awareness, Treatment, and Control of High Blood Pressure: A Population-Based Survey in Thai Nguyen, Vietnam. PLoS ONE 2013;8:e66792. https://doi.org/10.1371/journal.pone.0066792.

96 Hien HA, Tam NM, Tam V, Derese A, Devroey D. Prevalence, Awareness, Treatment, and Control of Hypertension and Its Risk Factors in (Central) Vietnam. Int J Hypertens 2018;2018:1–12. https://doi.org/10.1155/2018/6326984.

97 Van Minh H, Lan Viet N, Sinh CT, Hung PN, Ngoc NTM, Van Hung N, et al. May Measurement Month 2018: an analysis of blood pressure screening results from Vietnam. Eur Heart J Suppl 2020;22:H139–41. https://doi.org/10.1093/eurheartj/suaa049.

98 Minh HV, Poulter NR, Viet NL, Sinh CT, Hung PN, Ngoc NTM, et al. Blood pressure screening results from May Measurement Month 2019 in Vietnam. Eur Heart J Suppl 2021;23:B154–7. https://doi.org/10.1093/eurheartj/suab035.

99 Abu Hamad BA, Jamaluddine Z, Safadi G, Ragi M-E, Ahmad RES, Vamos EP, et al. The hypertension cascade of care in the midst of conflict: the case of the Gaza Strip. J Hum Hypertens 2022. https://doi.org/10.1038/s41371-022-00783-w.

100 Goma F, Syatalimi C, Tembo P, Mukupa M, Chikopela T, Kaluba L, et al. May Measurement Month 2019: an analysis of blood pressure screening results from Zambia. Eur Heart J Suppl 2021;23:B158–60. https://doi.org/10.1093/eurheartj/suab043.
