# Supplementary material for: Care Cascades for Hypertension in Low-Income Settings: A Systematic Review and Meta-Analysis
Source: Int J Public Health. 2023 Oct 12;68:1606428. doi: 10.3389/ijph.2023.1606428 (PMC10600349; doi:10.3389/ijph.2023.1606428)
Supplement: Supplementary file 7 [file DataSheet2.docx]

| **Low Income countries (LICs)** | **Lower-Middle Income countries (LMICs)** |
| --- | --- |
| Afghanistan | Angola |
| Burkina Faso | Algeria |
| Burundi | Bangladesh |
| Central African Republic | Benin |
| Chad | Bhutan |
| Congo, Dem. Rep | Bolivia |
| Eritrea | Cabo Verde |
| Ethiopia | Cambodia |
| Gambia, The | Cameroon |
| Guinea | Comoros |
| Guinea-Bissau | Congo, Rep. |
| Korea, Dem. People's Rep | Côte d'Ivoire |
| Liberia | Djibouti |
| Madagascar | Egypt, Arab Rep. |
| Malawi | El Salvador |
| Mali | Eswatini |
| Mozambique | Ghana |
| Niger | Haiti |
| Rwanda | Honduras |
| Sierra Leone | India |
| Somalia | Indonesia |
| South Sudan | Iran, Islamic Rep |
| Sudan | Kenya |
| **﻿**﻿Syrian Arab Republic | Kiribati |
| Togo | Kyrgyz Republic |
| Uganda | Lao PDR |
| Yemen, Rep. | Lebanon |
| Zambia | Lesotho |
|  | Mauritania |
|  | Micronesia, Fed. Sts. |
|  | Mongolia |
|  | Morocco |
|  | Myanmar |
|  | Nepal |
|  | Nicaragua |
|  | Nigeria |
|  | Pakistan |
|  | Papua New Guinea |
|  | Philippines |
|  | Samoa |
|  | São Tomé and Principe |
|  | Senegal |
|  | Solomon Islands |
|  | Sri Lanka |
|  | Tanzania |
|  | Tajikistan |
|  | Timor-Leste |
|  | Tunisia |
|  | Ukraine |
|  | Uzbekistan |
|  | Vanuatu |
|  | Vietnam |
|  | West Bank and Gaza |
|  | Zimbabwe |
|  |  |
